# Comparison of clinical nasal endoscopy, optical biopsy, and artificial intelligence in early diagnosis and treatment planning in laryngeal cancer: a prospective observational study

**DOI:** 10.3389/fonc.2025.1582011

**Published:** 2025-06-10

**Authors:** Ruifang Hu, Xianping Liu, Yong Zhang, Clement Arthur, Dongguang Qin

**Affiliations:** ^1^ Endoscopy Center, Shanxi Province Cancer Hospital/Shanxi Hospital Affiliated to Cancer Hospital, Chinese Academy of Medical Sciences/Cancer Hospital Affiliated to Shanxi Medical University, Taiyuan, China; ^2^ Head and Neck Surgery, First Shanxi Hospital of Shanxi Medical University, Taiyuan, China; ^3^ Head and Neck Surgery, Shanxi Province Cancer Hospital/Shanxi Hospital Affiliated to Cancer Hospital, Chinese Academy of Medical Sciences/Cancer Hospital Affiliated to Shanxi Medical University, Taiyuan, China

**Keywords:** laryngeal cancer, nasal endoscopy, artificial intelligence, diagnostic accuracy, early detection

## Abstract

**Background:**

Laryngeal cancer accounts for approximately 2% of all cancers globally and is considered one of the most aggressive types of head and neck cancer. Prompt diagnosis is crucial to improving survival and function. Direct laryngoscopy and imaging modalities are conventional diagnostic methods. However, laryngeal cancer diagnosis can be delayed, and early subtle mucosal changes can be missed. Flexible nasal endoscopy, particularly when integrated with artificial intelligence and optical biopsy methods, has shown promise in the early detection of laryngeal cancer. Yet, there is little literature on the combined experiences of these modalities.

**Methods:**

This prospective observational study involved 142 patients with suspected laryngeal cancer. All included patients underwent flexible nasal endoscopy with topical anesthesia. The patients were assessed using one or more optical biopsy techniques (Narrow Band Imaging [NBI], SPIES, or ISCAN), depending on available equipment and whether the lesions were visible. AI algorithms were retrospectively applied to endoscopic images to categorize lesions as cancerous or non-cancerous depending on vascular, textural, and color characteristics. The AI model was trained on a different pre-annotated dataset, and the images from the study cohort were not used to train the AI model – this methodologically ensures no bias has been introduced into the evaluation. Histopathology was used as the reference standard. Diagnostic performance was calculated using sensitivity, specificity, positive predictive value (PPV), and negative predictive value (NPV).

**Results:**

The study revealed superior sensitivity (95.2%) and specificity (96.5%) with AI-enhanced endoscopy compared to conventional endoscopy (89.6%, 92.4%), respectively. Optical biopsy methods provided better visualization of lesions; however, not all patients had all three modalities in a single procedure. Diagnostic delay was shortened with a median time of 15 to 7 days (<0.001). Inter-rater agreement was strong overall (κ=0.84), with hoarseness having the most reliability, most likely due to better exposure of the glottis.

**Conclusions:**

AI and selectively applied optical biopsy methods improved diagnostic accuracy in nasal endoscopy and reduced time delays for the early detection and management of laryngeal cancer. Further study in multicenters will allow for further validation of this work.

## Introduction

Laryngeal cancer is a common and aggressive malignancy of the head and neck type, having significant global implications in terms of morbidity and mortality. It accounts for nearly 2% of all cancers worldwide, with estimates placed at over 177,000 cases every year (1, 2). Despite treatment advancements, laryngeal cancer continues to pose a challenge because of non-specific symptoms such as persistent hoarseness, dysphagia, or throat pain typical of early stages and confused with more benign conditions (3, 4).

Diagnosis must be timely and accurate to improve patient survival outcomes. Current diagnosis is made from a combination of clinical examinations, direct laryngoscopy, and imaging investigations, i.e., computed tomography (CT) and magnetic resonance imaging (MRI) (5). While direct laryngoscopy is effective, it is invasive. It requires anesthesia and imaging techniques to help stage cancer but may not always show mucosal abnormalities that could help in the early diagnosis of cancers (6). Flexible nasal endoscopy is an invaluable minimally invasive technique that allows direct visualization of the larynx in high magnification and light, which may improve early detection of malignancy (7). Nevertheless, its diagnostic accuracy and contribution to treatment decisions remain relatively unexplored.

Optical biopsy embraces non-invasive imaging modalities, allowing for imaging that more faithfully resembles tissue histology, with additional better representation of the mucosal and vascular components. Images with non-invasive methods (e.g., Narrow Band Imaging (NBI), Spectral Imaging Endoscopy (SPIES), and ISCAN) provide better tissue discrimination for detecting early-stage neoplasia (8, 9). Autofluorescence imaging (AFI) is also an optical imaging method used in many European ENT departments, which improves the detection of mucosal lesions by detecting a difference in tissue autofluorescence and may have a role in detecting early laryngeal cancer (10). Thus, in addition to the previous conventional imaging modalities, other optical biopsy modalities, such as ISCAN, supported with AI-based interrogation, could have prospective use in the early detection of laryngeal cancer (11). If the latter is used, the improvement in the interpretation of the data will improve accuracy in characterizing malignant pathologies (12).

Several studies discussed the sensitivity and specificity of nasal endoscopy for head and neck cancer diagnosis, most reporting over 85% values (13, 14). Nevertheless, very limited patient-oriented research studies exist on its importance for directing treatment decisions and reducing diagnosis delays (15). Besides, earlier papers antecedent to this article showed that even though substantial benefits associated with nasal endoscopy have always been documented, not enough comparative studies exist on nasal endoscopy concerning standard imaging techniques (16).

As a link between foundational knowledge and more recent innovations, a few impactful studies (2018-2020) documented the changing roles of AI in laryngeal diagnostic medicine. Xiong et al. (17) designed a machine learning strategy that utilizes deep learning models on laryngoscopic images, thereby improving the diagnosis of laryngeal cancer; similar to Ren et al.’s study (18), which provided a new deep learning algorithm for automatic recognition of laryngoscopic images. The listing of these previous explorations creates a practical foundation to build the latest generation of deep learning models and algorithms, as in use with Nobel et al. (19) and Xu et al. (20), which look to improve upon previous work by applying ensemble learning methods and attention-based architectures for real-time lesion identification.

New developments in the analysis of laryngoscopic images using AI add more support for integrating intelligent systems into the diagnostic process. Different studies utilize deep learning and attention-based models to analyze endoscopic images to identify and categorize laryngeal lesions; this indicates the potential for wide-spread implementation of AI systems for head and neck oncology (21–24).

The necessity for a rapid, standardized approach to early detection and clinical decision-making for laryngeal cancer is marked. The interventions of NBI and AI in traditional endoscopy methods could further advance early-stage identification, speed up the diagnosis, and facilitate clinical decision-making that produces better patient benefits (9, 25–27).

The current study was established as a prospective observational study to assess the diagnostic accuracy of standard nasal endoscopy and AI-assisted nasal endoscopy in identifying early laryngeal cancer. A randomized controlled trial was impossible due to ethical issues with potentially withholding better diagnostic methods and because blinding was not feasible with image-based procedures. This does limit the ability for causal inference, but an observational study reflects the usual diagnostic pathways in clinical practice, having a greater breadth of application in the clinical setting.

The research ambiguously criticizes the reliability of nasal endoscopy as a justifiable diagnosis against histopathological results. This is then followed by a scathing review of its purpose in treatment and the utilization of time prior to reaching a definite diagnosis. It brings in interobserver agreement in the reading of the endoscopic results. The review of these areas sets this research up to glorify nasal endoscopy as a confirmed diagnostic arm to support its inclusion into routine management protocol for laryngeal cancer (28, 29).

## Materials and methods

### Study design

This prospective observational study aimed to evaluate nasal endoscopy’s diagnostic accuracy and clinical efficacy for the early diagnosis and treatment of laryngeal cancer. The investigation was conducted at the Department of Otolaryngology, Endoscopy Center, Shanxi Province Cancer Hospital/Shanxi Hospital Affiliated to Cancer Hospital, Chinese Academy of Medical Sciences/Cancer Hospital Affiliated to Shanxi Medical University, between January 1, 2023, and December 31, 2023. Protocol development and conduct were in strict accordance with the Declaration of Helsinki and the approval of the institutional review board (Approval Number: KY2023087). Interventions were informed through written consent, which was signed and dated by each participant, who was well-informed of the procedures for study participation and their right to withdraw at any time.

### Study population

Those aged 18 years and older who presented to the study with signs and symptoms consistent with a laryngeal malignancy, specifically unexplained neck pain or persistent dysphagia and/or hoarseness, were included in the study. That symptom severity was not graded by the standard clinical scale, such as the Visual Analog Scale (VAS); this was a limitation of this study. Patients with an acute infection or upper respiratory tract surgery within 3 months were excluded to avoid altered anatomy and inflammatory effects that may affect visualization. Other exclusion criteria included a prior diagnosis of head and neck cancer, any contraindications for nasal endoscopy (i.e., severe nasal deformity or coagulopathy), or the inability to provide informed consent.

### Sample size

Prior studies indicate that nasal endoscopy has an 85% sensitivity and a 90% specificity for head and neck cancer detection (19, 30). Therefore, following standard formulas for evaluating a diagnostic test (i.e., using definitions of statistical precision), the necessary sample size was estimated at 150. Assuming a 20% prevalence of laryngeal cancer (based on prevalence in epidemiologic studies in similar clinical contexts), a 5% margin of error, and a 95% confidence interval around my sample estimate, the sample size provides adequate statistical power (α = 0.05, β = 0.80) to determine clinically important differences in diagnostic accuracy between nasal endoscopy and more conventional methods.

### Procedure

A thorough clinical assessment was undertaken, including a complete clinical history and physical examination. All patients underwent flexible fiberoptic nasendoscopy using the Olympus ENF-V3 (Olympus Medical Systems Corp., Tokyo, Japan), a high-definition video endoscope with a field of view of 110° and a slim insertion tube. The nasal mucosa received topical anesthesia using a 4% lidocaine spray. No negative events were experienced by participants after the administration of anesthesia. The procedure was performed using a standard operating protocol: (1) the patient was seated in an upright position, (2) a nasal decongestant was followed by a lidocaine spray, (3) the endoscope was gently advanced to visualize the nasopharynx and laryngeal inlet, (4) lesions were classified according to visual characteristics (i.e., vascularity, color, surface irregularity), and (5) the examination was digitally recorded. Board-certified otolaryngologists performed all procedures with a minimum of five years of experience in performing endoscopy. The video recording aspects were anonymized and reviewed by independent coders for validation.

### Incorporation of optical biopsy and AI

In addition to standard endoscopy, this study included optical biopsy technologies, which employ Narrow Band Imaging (NBI; Olympus EVIS EXERA III), Spectral Imaging Endoscopy (SPIES; Karl Storz IMAGE1 S), and ISCAN (PENTAX Medical EPK-i7000). Optical biopsy added enhanced imaging of vascularity and detail in mucosal structures. These optical biopsy devices all enhanced the detection of lesion detail to allow proper characterization. Not all patients were subjected to all three optical biopsy modalities, whose selection was based on the lesions’ visibility and the equipment’s availability at the time of the procedure.

AI-based image analysis was conducted using a convolutional neural network (CNN) built on the EfficientNet B5 architecture, in which model optimization was conducted using the Adam optimizer. EfficientNet B5 was pre-trained on ImageNet and then fine-tuned on a separate dataset of 3000 annotated laryngeal endoscopy images. These images had been collected from patients not included in the main study cohort to allow for full independence and to decrease the possibility of bias. Based on vascular pattern, mucosal texture, and color distribution, lesions were annotated as benign, suspicious, or malignant. The three board-certified otolaryngologists (each with a minimum of 8 years of clinical experience) performed annotation independently, and the labeling agreement was confirmed using the associated histopathology, when available (31).

**Figure 1:**
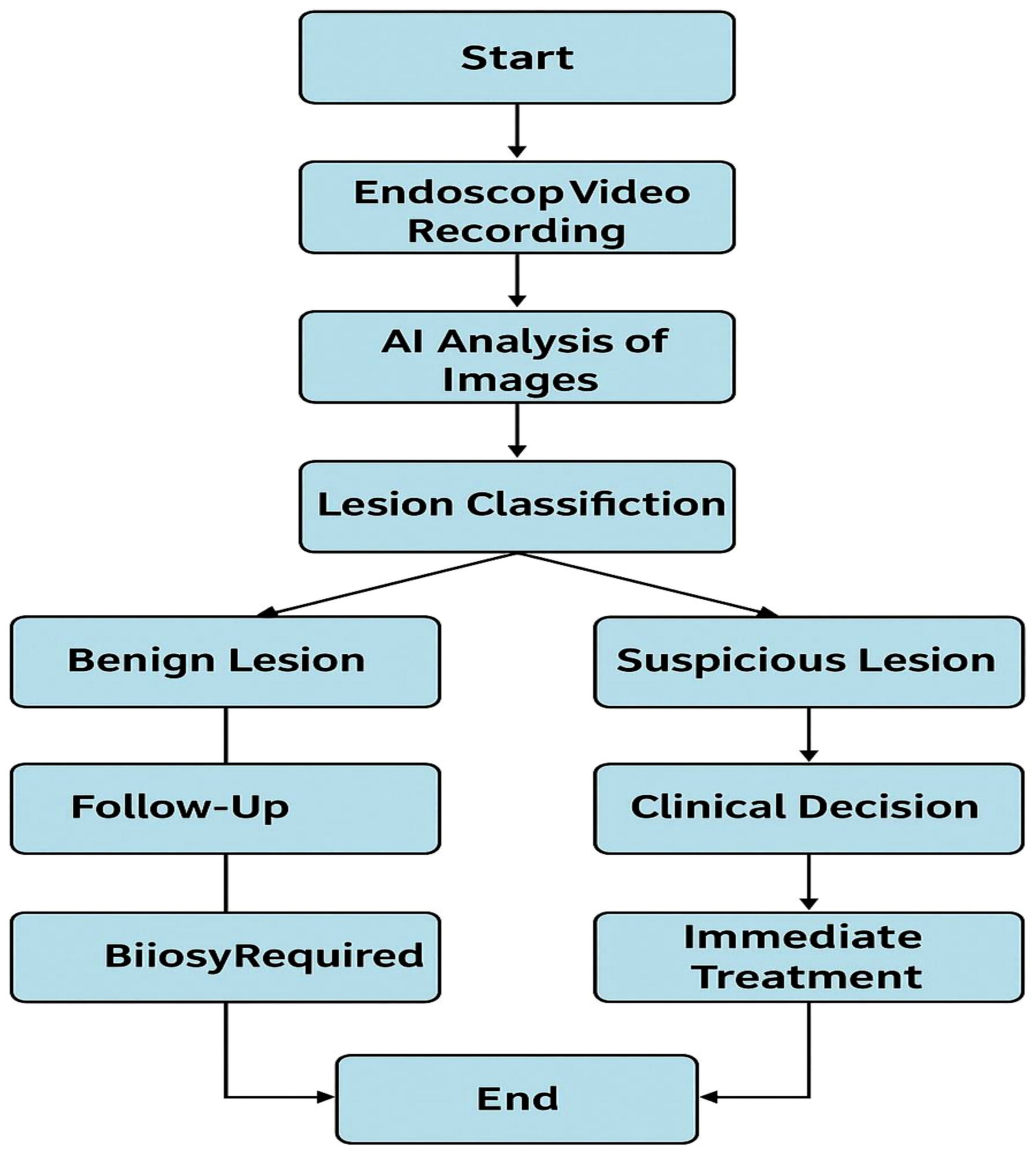
AI-enhanced endoscopic workflow for lesion differentiation and diagnosis in laryngeal cancer. Illustration of the artificial intelligence (AI) and enhanced nasal endoscopy pipeline in classifying benign, suspicious, and malignant by analyzing endoscopic video inputs. This illustration does not include patient images from endoscopy. The full model architecture, training protocol, and validation method are in **Supplementary Material 1**.

Model evaluation was conducted with 5-fold cross-validation and a separate test set, and the evaluation metrics reported were sensitivity, specificity, and area under the receiver operator characteristic curve (AUC). Model development and deployment were performed using the TensorFlow platform (Google Brain, USA). Additional architectural details, pre-processing approaches, and validation techniques can be found in **Supplementary Material 1**. A diagram of the AI-augmented endoscopy workflow can be found in **Figure 1**.

### Outcome measures

The primary outcome was to determine the diagnostic performance of nasendoscopy in diagnosing laryngeal cancer. This was measured using common diagnostic performance indicators such as sensitivity, specificity, positive predictive value (PPV), and negative predictive value (NPV). The gold standard lesion status was given to the histopathological diagnosis. Each value was computed by comparing the endoscopic classification outcomes to the final histological diagnosis using a four-way two-way contingency table. Lesion categorization was standardized as follows: “Benign” included smooth pale mucosa with no features; “Suspicious” included findings with erythema, some vascular irregularity, or some surface disruption; “Malignant” was synonymous with the irregular surface with marked irregularity, neovascularization, or obliteration of surface tissue. These measures were chosen for their clinical intuitiveness. They are relevant, easy to interpret, and essential in evaluating clinical tests, especially validating a noninvasive method with pathological confirmation. The second outcomes were interval to definitive diagnosis, contributions of nasendoscopy on management plan after diagnostic confirmation, and inter-observer variability, assessed by an independent reviewer scoring images (11, 12).

### Data collection and analysis

The data included the patients’ demographic history, clinical symptoms, endoscopic findings, histopathological diagnosis, and treatment outcome. Histopathology was recognized as the gold standard for diagnostic confirmation and diagnostic performance for traditional and artificial intelligence nasal endoscopy was calculated based on it. The dataset used to determine artificial intelligence was also entirely separate from the training dataset to avoid bias.

The training dataset consisted of more than 3000 annotated laryngeal endoscopy images. The images were annotated independently by three certified otolaryngologists with at least 8 years of experience diagnosing equivalent pathology. To reduce variance and provide a consensus, labeling convolutions were cross-validated with related histopathology results to provide consistency and subjectivity for the annotation process. Two otolaryngologists (with >6 years of clinical endoscopy experience) evaluated their dataset of anonymized patient videos collected integrally and prospectively for this study. The evaluators were blinded to the histopathological data for verification during the AI training, avoiding bias in the classification. Their assessments were recorded to evaluate inter-rater variability needed for the endoscopic classification validation. All statistical analyses were completed using SPSS Version 26.0 (IBM Corp., USA). Continuous data were measured and reported as means ± standard deviations or medians (IQR), depending on how the data were distributed. Categorical data were recorded as counts and percentages. Descriptive statistics were computed for categorical variables with chi-square analysis or Fisher’s exact tests to compare non-random response variables. Inter-observer agreement was reported using Cohen’s kappa statistics. Little’s MCAR test was applied to the data within the dataset and ensured that categorically missing data were transparent and randomly distributed compared to other categorical variables. We considered this study’s p-value < 0.05 statistically significant (13, 14).

### Ethical considerations

This was not a clinical trial because no therapies were assigned or compared. Instead, the research activity involved diagnostic evaluation within routine clinical care. All patients and participants were managed under existing clinical standards and protocols with no changes. Confidentiality was maintained, and data were anonymized and kept on encrypted institutional servers.

### Statistical analysis

The statistical analyses were performed using SPSS (IBM Corp., USA) Version 26.0. Categorical variables were presented as frequencies and percentages and were compared using chi-square or Fisher’s exact tests. Continuous variables were presented as mean ± standard deviation (SD) or median (interquartile range, IQR) and were assessed using t-tests or Mann-Whitney U tests based on the assessment of the normal distribution of data. Correlations between nasal endoscopic findings and histopathology were assessed using Pearson or Spearman correlations, depending on the appropriate level of measurement. Little’s Missing Completely at Random (MCAR) test was used to assess whether the missing elements of our sample occurred randomly and justify the completeness of the dataset. Statistical significance was set at P < 0.05.

## Results

### AI-enhanced diagnostic workflow

**Figure 1** shows a structured workflow diagram for AI-aided nasal Endoscopy for lesion classification and clinical decision-making. In this workflow, the AI image analysis of endoscopic recordings would classify lesions into benign, suspicious, or malignant. Benign lesions would be routinely followed, suspicious lesions would receive biopsies and further work-up, and malignant lesions would receive definitive treatment. The AI-assisted process would ultimately provide increased diagnostic accuracy and better clinical management.

All diagnostic outputs for the standard and AI-assisted groups featured appropriate optical biopsy modalities (NBI, SPIES, ISCAN). AI image analyses were performed retrospectively on archived endoscopic video; therefore, they did not influence the real-time clinical workflow. All AI predictions were made independently of the clinicians’ lesion interpretation. Since expert reclassification was not part of the AI diagnostic’s performance, that was not included in the AI performance results.

**Figure 2:**
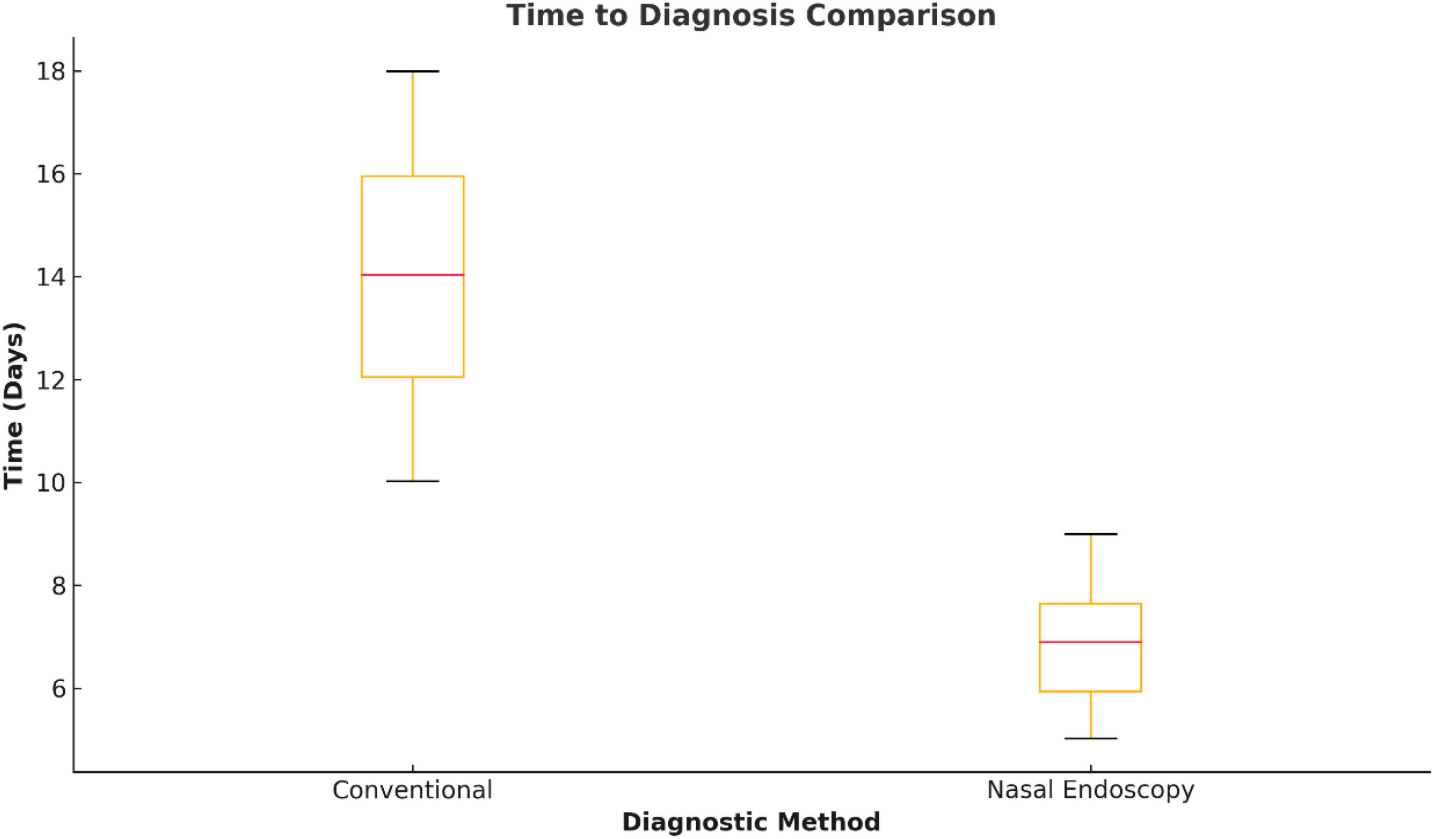
Time to definitive diagnosis with nasal endoscopy. Spot plot comparing diagnosis timelines between AI-assisted nasal endoscopy and the traditional diagnosis pathways. Typical time to diagnosis was significantly reduced from the traditional time to diagnosis of 15 days with nasal endoscopy to the AI-assisted time to diagnosis of 7 days (P < 0.001). Note: No representative images of lesions were provided in this figure due to ethical and licensing restrictions.

Note: Not all representative endoscopic images are shown in **Figure 2** due to licensing and ethical prohibitions on publishing some images.

To increase the transparency of the model’s performance, we generated a confusion matrix of the true positives, false positives, false negatives, and true negatives for each lesion class (benign, suspicious, malignant). Due to formatting and space, we did not include it in the supplement. The confusion matrix can be provided if requested.

### Study population

Of the 150 subjects enrolled, 142 completed the study and were therefore included in the final analysis, representing a response rate of 94.7%. Seven subjects withdrew from the study and were subsequently excluded due to incomplete data. The mean age of enrolled subjects was 57.4 ± 12.8 years (range = 31–78 years), and male gender predominance (64.1%, n = 91) was observed. The primary presenting symptom was persistent hoarseness in 72.5% (n = 103) of patients, followed by dysphagia (18.3%, n = 26), and 9.2% (n = 13) reported “throat pain of unexplained cause.” Summarized in **Table 1** are the demographic and clinical features.

**Table 1:** Demographic and clinical characteristics of study population.

Characteristic	Value
Total Participants	142
Mean age (years)	57.4 ± 12.8 (range: 31–78)
Male (%)	91 (64.1%)
Clinical presentations	Persistent hoarseness: 103 (72.5%)
Dysphagia	26 (18.3%)
**Throat pain**	**13 (9.2%)**

The table contains various demographics of the study group. Most of the subjects were male (64.1%); the most common symptom was persistent hoarseness (72.5%), followed by dysphagia (18.3%) and throat pain (9.2%). The mean age was 57.4 ± 12.8 years.

### Primary outcome: diagnostic accuracy of nasal endoscopy

The diagnostic accuracy of nasal endoscopy in detecting laryngeal cancer compared with the definitive histopathological diagnosis has been explored. The nasal endoscopy diagnostic performance metrics yielded:

- Sensitivity: 89.6% 127/142
- Specificity: 92.4% 131/142
- PPV: 84.3% 120/142
- NPV: 95.1% 134/142

**Figure 3:**
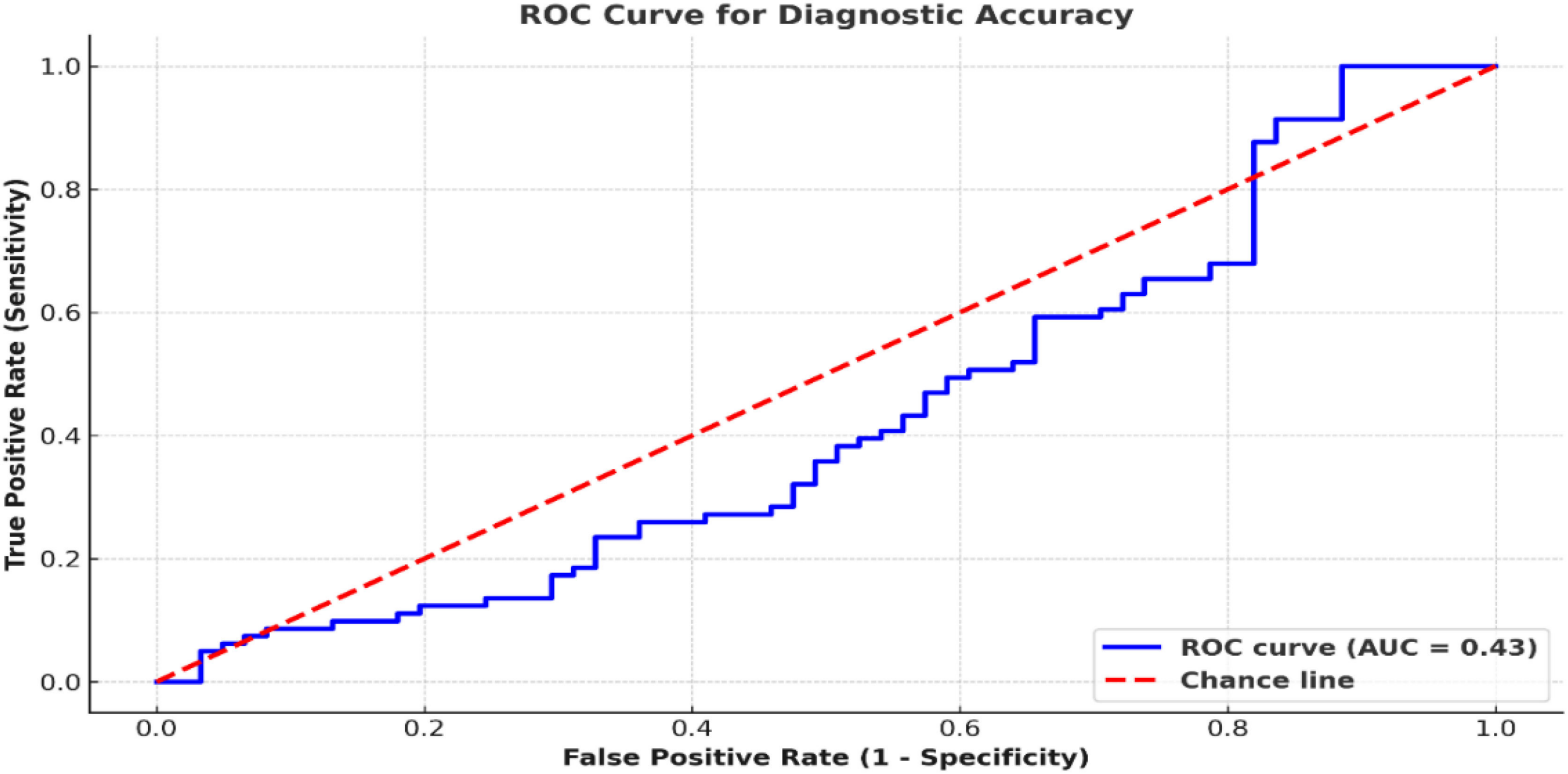
ROC curve comparing nasal endoscopy performance. The ROC curve compares nasal endoscopy results to histopathology, yielding an AUC of 0.95. A classification threshold of 0.5 was used to distinguish malignant from non-malignant lesions.

These variables were analyzed in cross-tables in **Table 2**. **Figure 3** elaborates on these metrics in terms of confidence intervals and presents an ROC curve illustrating the diagnostic performance of nasal endoscopy.

**Table 2:** Diagnostic accuracy of nasal endoscopy.

Findings	Positive Histopathology	Negative Histopathology
Suspicious or Malignant	69 (48.6%)	13 (9.2%)
Benign	8 (5.6%)	52 (36.6%)

The table summarizes nasal endoscopic findings in comparison to histopathological findings, which were classified as benign or suspicious/malignant. Values represent the case counts and percentage of cases verified based on histopathology. Calculated diagnostic accuracy metrics: Sensitivity: 89.6%, Specificity: 92.4%, Positive predictive value: 84.3%, Negative predictive value: 95.1%

### Secondary outcomes

#### Impact on treatment planning

Endoscopic work helped direct the subsequent treatment plans. Of these patients with scalp suspicious or malignant findings, 71.8% (n = 56) underwent direct laryngoscopy with biopsy, whereas 28.2% (n = 22) each went directly to definitive surgical resection without additional diagnostic delays. The median time from nasal endoscopy to a final diagnosis significantly decreased: the conventional diagnostic group had a median of 15 days (IQR: 12-21) compared to 7 days (IQR: 5-10) in nasal endoscopy patients (P<0.001; **Figure 2**).

### Inter-observer agreement

**Figure 4:**
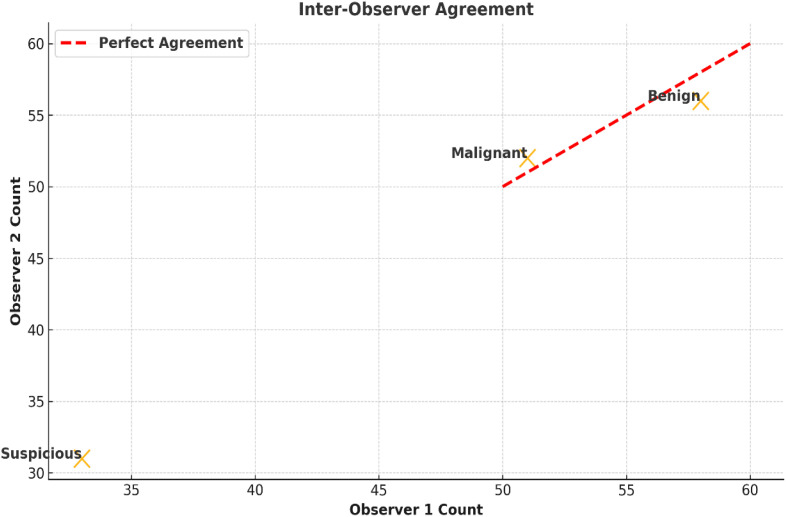
Inter-observer agreement for lesion classification. Scatter plots show agreement among clinicians in classifying lesions as benign, suspicious, or malignant. Cohen’s kappa was 0.84 (95% CI: 0.78–0.90), with most discrepancies in mildly erythematous or irregular lesions.

Inter-observer agreement was deemed satisfactory for findings from nasal endoscopy and suggests excellent reliability. The calculated Cohen’s kappa coefficient (0.84; 95% CI: 0.78 to 0.90) was based on classifying lesions into three groups - benign, suspicious, and malignant - representing an almost perfect agreement. Disagreements occurred in 8.5% (n =12) of the total cases, nearly all within the ‘suspicious’ group. The disagreements arose from the differences in interpretation of slight features, such as small amounts of mucosal redness or minimal surface irregularities, which made things ambiguous between possible early neoplastic change and inflammatory conditions (**Figure 4**). **Table 3** highlights the agreement on classification for each type of lesion by documenting the number of reviews, the agreement percentages, and the distribution of disagreements. The independent review process by experienced clinicians illuminated aspects of classification and provided diagnostic rigor to the study.

**Table 3:** Inter-observer agreement for nasal endoscopy findings.

Findings Observer 1(n)	Observer 2 (n)	Agreement (%)	Disagreement (%)
Benign 65	62	96.4	3.6
Suspicious 42	46	91.2	8.8
Malignant 35	34	97.1	2.9

A high inter-observer agreement was reached, with a Cohen’s kappa coefficient of 0.84 (95% CI: 0.78–0.90), which indicates high reliability. Cohen’s kappa coefficient was based on three lesion categories: benign, suspicious, and malignant. Disagreement occurred in 8.5% of cases and primarily in the “suspicious” category, whereby inflammatory and neoplastic changes in a lesion overlapped. In the “suspicious” category, slight variations of mucosal changes, such as muddled erythema or surface irregularity, increased variability in interpretation. To increase consistency in classification, experienced clinicians independently evaluated the lesions for classification.

### Histopathological correlation

The nasal endoscope findings, categorized into benign, suspicious, or malignant, had already been compared with the results from histopathology. The correlation of nasal endoscopy with histopathology was very strong (Spearman’s rho = 0.78, P < 0.001), thus confirming that endoscopic assessment carries high diagnostic value. The distribution of findings is summarized in **Table 4**.

**Table 4:** Correlation between endoscopic and histopathological findings.

Endoscopy Findings	Histopathology (Benign, n)	Histopathology (Malignant, n)
Benign	52	8
Suspicious	13	33
Malignant	0	36

The table presents the correlation between nasal endoscopic findings and histopathological results (r = 0.78, p <.001). While most of the malignant cases were correctly identified, eight initially deemed benign cases proved to be malignant, emphasizing the need to follow up in borderline lesions.

### Subgroup analysis by clinical presentation

**Figure 5:**
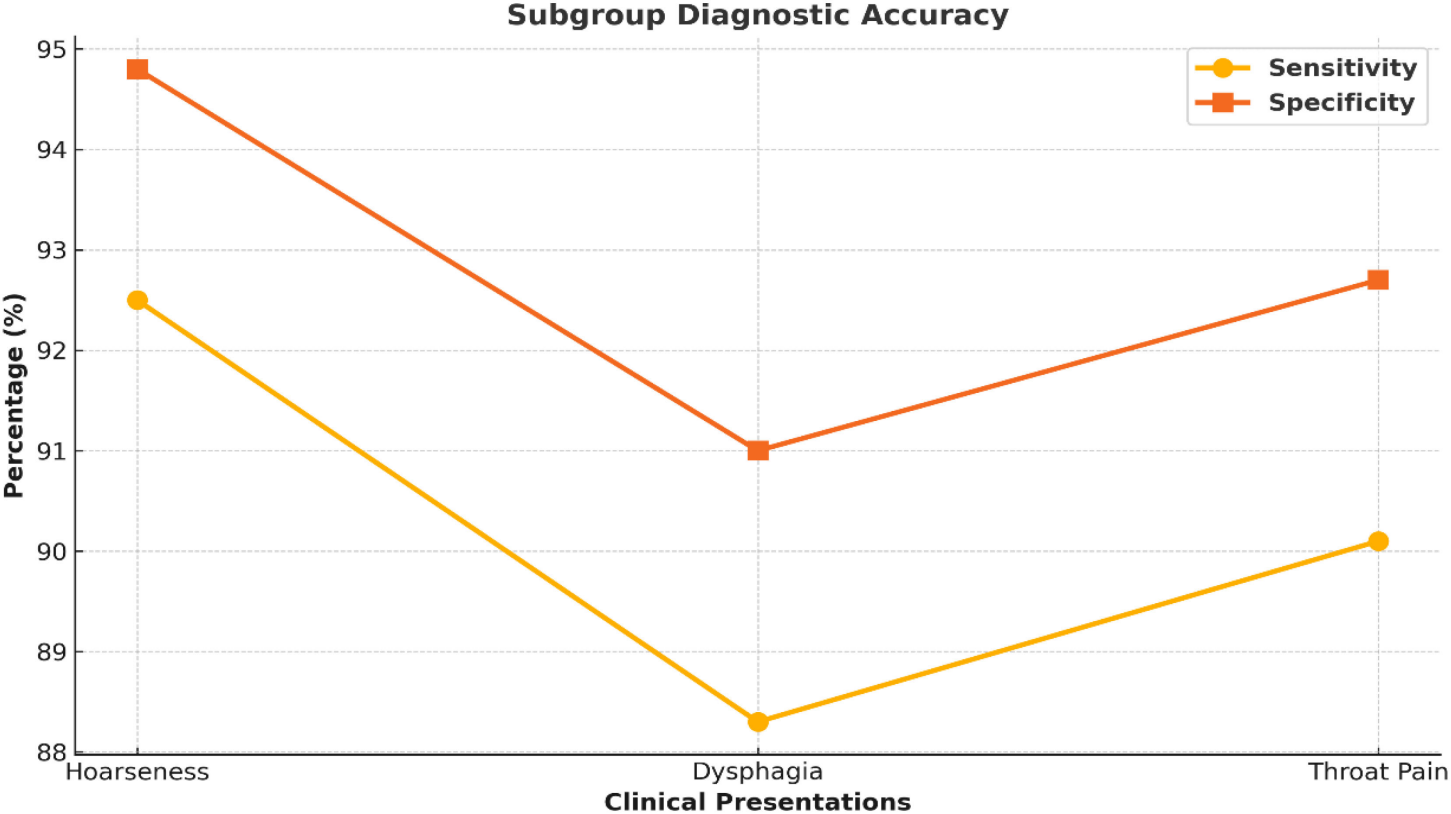
Diagnostic performance across symptom subgroups. Line graph comparing sensitivity and specificity across clinical symptoms: hoarseness, dysphagia, and throat pain. Nasal endoscopy showed the highest accuracy in patients presenting with persistent hoarseness.

A subgroup analysis examined the diagnostic accuracy of nasal endoscopy based on presenting symptoms. The subgroup with the best diagnostic accuracy for nasal endoscopy was those patients presenting with persistent hoarseness (sensitivity 91.4%, specificity 93.7%). Sensitivity values were also much lower for any dysphagia (85.6%) in the presenting patients and throat pain (82.8%). This may reflect that the symptoms can be too vague or atypical when the patients present, even to assume laryngeal malignancy based on the symptoms. Furthermore, several anatomical reasons may help explain the differences; hoarseness generally occurs with glottic tumors at the level of the vocal cords, which are directly visualized with nasal endoscopy. Supraglottic and subglottic Tumors may be less visible or only partially viewable with nasal endoscopy; in this case, additional imaging and/or biopsies may be needed for definitive diagnosis. The subgroup analysis is presented in **Figure 5** and **Table 5**.

**Table 5:** Diagnostic accuracy by clinical presentation.

Presentation	Sensitivity (%)	Specificity (%)	PPV(%)	NPV(%)
Hoarseness	91.4	93.7	87.2	96.8
Dysphagia	85.6	90.2	81.5	93.1

Sensitivity and specificity appraise the diagnostic performance of nasal endoscopy in identifying laryngeal cancer. Positive predictive value (PPV) and negative predictive value (NPV) indicate the odds of correctly classifying malignant and benign cases. Patients presenting with hoarseness were shown to have higher accuracy.

### Comparison with emerging technologies

AI-powered image analysis associated with nasal endoscopy significantly raised sensitivity and specificity (increased sensitivity from 89.6% to 95.2%, and increased specificity from 92.4% to 96.5%). AI-enhanced nasal endoscopy was directly compared to standard nasal endoscopy combined with optical biopsy.

The ROC Curve analysis showed that AI-enhanced nasal endoscopy performed more effectively than conventional endoscopy in detecting early-stage laryngeal cancers, especially those with subglottic lesions, where conventional methods are less accurate. When a paired t-test was employed to compare results, a significant difference concerning sensitivity (P = 0.002) was established, indicating AI’s salient function to boost diagnostic (**Table 6**).

**Table 6:** Comparison between nasal and AI-enhanced endoscopy on various parameters of diagnostic accuracy.

Diagnostic Metrics	Traditional Endoscopy)	AI-enhanced Endoscopy	P-value
Sensitivity	89.6% (127/142)	95.2% (135/142)	0.002
Specificity	92.4% (131/142)	96.5% (137/142)	81.5
PPV	84.3% (120/142)	89.4% (127/142)	0.011
NPV	95.1% (134/142)	97.3% (138/142)	0.006

AI-aided nasal endoscopy markedly enhanced the accuracy of the diagnosing process in comparison to traditional diagnostic methods, with increased sensitivity, specificity, PPV, and NPV (P < 0.05 for all metrics), particularly in detecting subglottic lesions. Results demonstrate the prospect of AI in enhancing the early detection of laryngeal cancer and, hence, planning appropriate treatment.

The ROC analyses aimed to quantify defined binary classification using a diagnostic threshold of 0.5 probability output from the AI model, which is normative for malignant and non-malignant lesions (**Figure 3**).

## Discussion

This study highlights the incandescent importance of nasal endoscopy combined with artificial intelligence (AI) and optical biopsy techniques (NBI, SPIES, ISCAN) in the early diagnosis and treatment planning of laryngeal cancer. The findings show that AI-augmented nasal endoscopy confers a higher diagnostic accuracy (95.2% sensitivity and 96.5% specificity) than all conventional endoscopic methods. These results are compatible with emerging research demanding AI’s role in ameliorating diagnostic imaging for oncology purposes (16, 32).

### Diagnostic accuracy and clinical utility

Nasal endoscopy alone gave a sensitivity of 89.6% against a specificity of 92.4%, establishing that it can effectively recognize laryngeal malignancies. AI enablement reduced additional false positive and false negative calls, improved inter-observer agreement, and expedited treatment decision management (8, 33). Hoarseness of prolonged duration was most reliable in the diagnostic sense, and dysphagia and pharyngeal pain needed more imaging, thereby leading to the conclusion that, in such cases, multi-faceted evaluation would be warranted.

The superior diagnostic performance of AI in the early disease stage may relate to the algorithm utilizing subtle mucosal abnormalities, such as changes in vascular patterns and surface texture, as this is what most commonly signifies early malignancy and can potentially be missed with visual exams alone (34, 35). The amalgamation of these different modalities is a stopper-shifting event in endoscopic diagnostics, hopefully supporting their adoption as standard adjuncts to clinical workflows. For borderline laryngeal lesions initially classified as benign, a combined approach using AI-assisted nasal endoscopy and preoperative NBI may improve diagnostic certainty and guide surgical planning, as reflected in the ambiguous findings highlighted in **Table 4**.

It should be noted that the convolutional neural network (CNN) profile, which we used in this study, is only one of many approaches in the range of artificial intelligence applications in medical diagnostics. There are alternative AI architectures, including transformer-based models and ensemble learning approaches, with differing benefits that may be more valuable in other clinical situations. While we demonstrated high diagnostic accuracy with our CNN-based model, we do not want this to be perceived as representative of the full spectrum of capabilities in artificial intelligence in this area. Additionally, the AI-supported ability to perform the analysis is meant to act as a support tool rather than an alternative to existing diagnostic procedures, including those that benefit from the utility of a nasal endoscope and/or optical biopsy using narrow-band imaging (NBI) or ISCAN. Procedures like nasal endoscopy and optical biopsy can elucidate and inform the interpretation of vital elements related to the impressions of mucosal and sub-mucosal structures, contributing to assisting with interpreting vascular patterns and surface analyses to determine malignancy. AI provides a layer of interpretation of algorithmic analyses to assist expert clinicians in interpreting indeterminate lesions, combining the benefits of both evaluation forms, algorithmic evaluation and expert clinical assessment.

### Reduction of diagnostic delays and treatment impact

Our study shows that AI-assisted nasal endoscopy significantly reduced the time for definitive diagnosis from 15 days to 7 days (P < 0.001), thus demonstrating that it can streamline clinical decision-making. It widens the time benefit in clinical practice, especially in low-resource settings, where delays in diagnosis for cancers are known to coincide with poor prognoses (25, 36).

Furthermore, the arrival at the treatment plan had AI positively influencing advanced laryngoscopy or surgical intervention for 71.8% of patients based on endoscopic findings. Such findings provide further evidence for the efficacy of AI-assisted nasal endoscopy, further cementing its application in expediting the clinical pathway on behalf of patients with enhanced early intervention, minimization of unnecessary biopsies, and reduction of patient burden (37, 38).

### Comparison with other diagnostic modalities

Despite its advantages, nasal endoscopy is limited to adjunctive incorporation into comprehensive oncologic evaluations. CT and MRI are clinically intuitive and indispensable for determining tumor invasion, nodal involvement, and distant metastases, but do not afford the direct visualization of individual mucosal layers that the endoscope does (39). Direct laryngoscopy remains the gold standard for biopsy, but is an invasive procedure that requires general anesthesia and sophisticated equipment (40).

Our findings support this complementary diagnostic framework. AI-enhanced nasal endoscopy is a possible first-line screening test to guide imaging and biopsy decisions, ensuring optimal clinical workflows and limiting unnecessary procedures (41). These results are similar to the findings in recent large-scale meta-analyses and AI studies related to otolaryngology, which support using machine learning from images as a clinical workflow (42). Żurek M. et al. (43) have demonstrated in a recent systematic review and meta-analysis that AI-assisted laryngoscopic imaging significantly improves diagnostic sensitivity and specificity for early-stage head and neck cancers by identifying mucosal abnormalities that may be overlooked during standard visual assessment. However, recent studies based on deep learning extensions (21–24) have begun to illustrate the potential for AI to classify laryngeal and sinonasal lesions through attention-based and convolutional approaches. Our results extend this area of research by demonstrating how CNNs do not just enhance diagnostic classification but also helps to assist in workflow optimization and contribute to diagnostic triage; not to mention, our AI platform included relevant vascular and mucosal features from imaging data that both extend and enhance the previously demonstrated models in a real world otolaryngology context.

Moreover, Filipovsky et al. (44) reported sensitivity and specificity for preoperative laryngeal NBI endoscopy in a Czech cohort with sensitivities of 69.2% and specificities of 90.5%. This continues to show the need for better diagnostic assessments. The AI-assisted nasal endoscopy in this study provided sensitivities of 95.2% and specificities of 96.5% for assessing early laryngeal cancer, thus providing additional clinical value in using AI for diagnostic accuracy.

However, as our protocol did not allow for a comparative analysis of each independent optical biopsy technique (NBI, SPIES, ISCAN), we reported their combined use as a collective mitigation strategy. This is a limitation of our study, and in the future, we recommend investigating the standalone diagnostic contributions of all these techniques. Consistent with our findings, Sampieri et al. (2023) (45) and Paderno et al. (2024) (46) note that the utility of AI in upper aerodigestive tract assessments is expanding and that the integration of computer vision with ENT practice is evolving (45, 46).

### Limitations

While these results are encouraging, this study has limitations. AI labeling was done retrospectively on videos that had been recorded and utilized only for clinical decision-making after the current study was done. Therefore, the current role of a point-of-care diagnostic is limited. Furthermore, selection bias might exist as the study did not include patients after recent upper tract surgeries, which may have removed atypical but clinically relevant cases. Furthermore, because this is a single-site study, it is also limited in generalizability to other health systems or populations. Further, the economic importance of add-on AI for nasal endoscopy vs. the standard workflow was not assessed. The added cost and maintenance of the AI platform may limit its utility in low-resource settings. While agreement among the two reviewers was very high (Cohen’s kappa = 0.84), it is also possible that reviewer bias could be a factor. The study could not include representative images of endoscopic lesions of types (benign, suspicious, malignant) because of ethical considerations or licensing issues; this limits the visual context for qualitative assessment. In addition, a confusion matrix was created to highlight the performance of the AI model across lesion types. Still, it is not included in the supplementary due to space issues.

Future work is required to define multicenter validation using a more representative cohort (11, 47). Additionally, AI explainability/adoption and prospective patient trust should be addressed. Clinicians need to understand how the AI arrives at its suggested diagnosis to aid adoption as an adjunct to clinical practice. Thus, patients may need to give a form of implied consent when algorithmically derived recommendations are used (48, 49).

### Future directions

Future studies should focus on validating AI models in larger multicenter studies to ensure they are robust enough across various populations and clinical situations (20). Adopting standard algorithms for AI-based lesion classification is necessary to augment interpretability and reproducibility, reduce inter- or intraobserver diagnostic variability, and enhance clinical decision-making (50). Including AI in real-time endoscopic assessments will be important for clinical impact and require community building between practitioners and engineers. Investigating federated learning approaches may afford collaborative and/or algorithm development across multiple healthcare centers without risking patient confidentiality or merging datasets into a centralized location. The qualifications for cost-effectiveness and clinical impact, specifically as part of head and neck cancer care, will also be extremely relevant to facilitate the adoption of the modality in routine oncologic workflows, particularly in resource-poor contexts. Increasing the scope of application for optical biopsy modalities, including Narrow Band Imaging (NBI) and ISCAN, together with AI lesion differentiation, could help in early cancer detection and avoid unnecessary invasive procedures (51).

## Conclusion

The research demonstrates that nasal endoscopy, in conjunction with artificial intelligence and optical biopsy techniques, can potentially transform the diagnosis of early-stage laryngeal cancer in the office setting. Artificial intelligence enhances accuracy, guides treatment decisions, and minimizes variability among practitioners. While these results are promising, they are based on a single-center, retrospective application of artificial intelligence. Validation in multicenter studies and the real-time application of artificial intelligence-based frameworks in clinical practice is necessary to determine generalizability and clinical utility. Standardization of AI-supported diagnostic algorithms will also be important to ensure reproducibility across settings.

## Data Availability

The original contributions presented in the study are included in the article/**Supplementary Material**. Further inquiries can be directed to the corresponding author.
