# Supplementary material for: Comparison of clinical nasal endoscopy, optical biopsy, and artificial intelligence in early diagnosis and treatment planning in laryngeal cancer: a prospective observational study
Source: Front Oncol. 2025 Jun 10;15:1582011. doi: 10.3389/fonc.2025.1582011 (PMC12185544; doi:10.3389/fonc.2025.1582011)
Supplement: Supplementary file 1 [file DataSheet1.pdf]

Supplementary Material – AI Model Description

1. Algorithm Type:

A convolutional neural network (CNN) based on a modified ResNet-50 architecture was employed to automatically classify lesions in nasal endoscopy images.

2. Training Data:

The model was trained using a dataset of 3,200 anonymized laryngeal endoscopic images annotated by board-certified otolaryngologists. An 80:20 train-test split was used, and model performance was evaluated through 5-fold cross-validation.

3. Platform and Tools:

Model development and testing were conducted using Python 3.8 with TensorFlow 2.6.0 in a GPU-accelerated environment using NVIDIA CUDA drivers.

4. Input Features:

Images were resized to 224 × 224 pixels prior to input. Key diagnostic features extracted included vascular pattern irregularities, mucosal surface texture, and color variations typical of benign, suspicious, and malignant lesions.

5. Output Classes:

- Benign
- Suspicious
- Malignant

6. Validation and Performance Metrics:

On the test dataset, the model achieved:

- Sensitivity: 95.2%
- Specificity: 96.5%

- Area Under the ROC Curve (AUC): 0.972

- F1 Score: 0.91

7. Limitations:

The AI model was applied retrospectively to stored endoscopic images. This study did not perform real-time clinical integration. Future work should explore prospective real-time applications and federated learning for broader validation.

Supplementary Material 1 provides further technical specifications of the AI model, including its architecture, training-validation design, and performance metrics.
